# Ongoing toxin-positive diphtheria outbreaks in a federal asylum centre in Switzerland, analysis July to September 2022

**DOI:** 10.2807/1560-7917.ES.2022.27.44.2200811

**Published:** 2022-11-03

**Authors:** Jacob Kofler, Alban Ramette, Patricia Iseli, Lea Stauber, Jens Fichtner, Sara Droz, Annina Zihler Berner, Anna Bettina Meier, Michelle Begert, Sabine Negri, Anne Jachmann, Peter Michael Keller, Cornelia Staehelin, Barbara Grützmacher

**Affiliations:** 1 Cantonal Medical Office, Health Directorate of the Canton of Bern, Bern, Switzerland; 2 Institute for Infectious Diseases, University of Bern, Bern, Switzerland; 3 Staff Medical Service, Bern University Hospital and University of Bern, Bern, Switzerland; 4 Staff Medical Service, Federal Asylum Center, Bern, Switzerland; 5 Department of Infectious Diseases, Bern University Hospital and University of Bern, Bern, Switzerland

**Keywords:** *Corynebacterium diphtheriae*, diphtheria, epidemiology, public health, genomic sequencing

## Abstract

Two diphtheria outbreaks occurred in a Swiss asylum center from July to October 2022, one is still ongoing. Outbreaks mainly involved minors and included six symptomatic respiratory diphtheria cases requiring antitoxin. Phylogenomic analyses showed evidence of imported and local transmissions of toxigenic strains in respiratory and skin lesion samples. Given the number of cases (n = 20) and the large genetic diversity accumulating in one centre, increased awareness and changes in public health measures are required to prevent and control diphtheria outbreaks.

In October 2022, an increase in diphtheria was noted in Europe by the European Centre for Disease Prevention and Control (ECDC) and the World Health Organization Regional Office for Europe (WHO/Europe) [1,2]. According to WHO/Europe, by the end of August 2022, 144 cases were reported, while the average number of notified cases in the previous 10 years ranged between 32 and 82 per year. About two thirds of the 2022 cases presented with cutaneous diphtheria, predominantly caused by *Corynebacterium diphtheriae*. However, respiratory cases also occurred, including one fatal case. The majority of cases were reported among asylum seekers or refugees, primarily from Afghanistan. Here we report findings of investigations of two diphtheria outbreaks in a Swiss asylum center from July to September 2022. The second outbreak is still ongoing at the time of publication.

## Outbreak chronology and implementation of public health measures

### First outbreak

**Figure 1:**
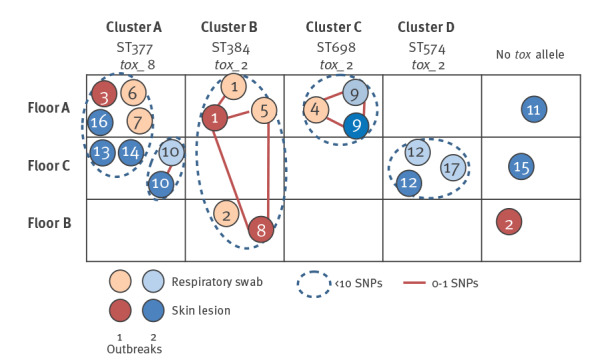
Genomic similarities among *Corynebacterium diphtheriae* isolates obtained from residents during two outbreaks at an asylum centre, Switzerland, July–September 2022 (n = 17)

On 21 July 2022, we identified a first case of toxin-positive diphtheria in a teenage asylum seeker with cutaneous lesions in our centre in Bern (Figure 1). The patient was treated according to Swiss guidelines (amoxicillin 1 g three times per day over 14 days) and was isolated [3]. Asymptomatic respiratory carriage of the same strain as from the skin was evidenced in a pharyngeal sample (Case 1). Later, *Corynebacterium diphtheriae* was detected in swabs from skin lesions of an asylum seeker in their 20s and a teenager 5 days later (Cases 2 and 3). Demographical, clinical and microbiological data of cases can be found in the Supplementary Table S1, including age, country of origin, date of microbiological tests and results, antibiotic therapy, as well as travel routes.

SNPs: single nucleotide polymorphisms.

Sequencing data were available for 17 of 20 cases. Cases were distributed across three floors and are identified by numbers 1 to 17 (Supplementary Table S1). The colour code indicates the association with first or second outbreaks, and specimen types. Cases and isolates are grouped by columns consisting of multi-locus sequence typing (MLST) and core genome MLST (cgMLST) clusters. Further insights on genomic relatedness are provided by displaying pairwise core genome single nucleotide polymorphisms relationships (Supplementary Table S2).

To stop the accumulation of cases, outbreak prevention measures were started on 26 July 2022 throughout the institution, with mandatory use of face masks and a stop of new allocations of asylum seekers to the centre. Pharyngeal swabs were taken from all residents and healthcare professionals of floor A, a floor dedicated to minors where Cases 1 and 3 were staying, and all received prophylactic antibiotic treatment consisting of 500 mg azithromycin once daily for 3 days. Immunisation with a diphtheria toxoid-containing vaccine (Boostrix Polio, GlaxoSmithKline plc, London, United Kingdom) was offered and strongly recommended. Approximately 90 to 95% were vaccinated over 2 days, on 26 and 27 July. The screening identified four additional cases of toxin-positive pharyngeal carriage (Cases 4 to 7, Supplementary Table S1) among residents of floor A while 88 people tested negative. All positive cases were treated with azithromycin 500 mg once daily for 3 days (as per Swiss guidelines for asymptomatic carriers) and isolated.

Remarkably, Case 2 who resided on floor B, had a toxin-positive result in the pharyngeal swab associated with a *C. diphtheriae* isolate, while their wound swab was toxin-negative and associated with a non-toxigenic *C. phoceense* isolate. As respiratory transmission by a resident of floor A carrying a toxin-positive strain could not be excluded, the same public health measures as on floor A were also implemented on floor B, and 136 residents were tested within the remaining entire facility. No other pharyngeal carriage could be identified in residents on floor B nor in the rest of the centre, but one additional resident was identified with a toxin-positive wound infection. None of the residents with toxin-positive results reported respiratory symptoms.

Genome sequencing was performed on all cultures to assess the clonal origin of this outbreak in order to adapt outbreak control measures. In total, eight cases were treated in the first outbreak. On 8 August the outbreak measures were lifted.

### Second outbreak

The second outbreak started on 1 September and is still ongoing at time of publication of this article. We present sequencing and epidemiological data on cases until 28 September. Some further information on cases until 28 October is presented in Supplementary Table S1 and in the Discussion. The first case of cutaneous diphtheria was confirmed during the entry screening of a teenage asylum seeker allocated to floor A (Case 9, Figure 1 and Supplementary Table S1).

Outbreak control measures during the second outbreak included antibiotic treatment of cases, isolation and screening pharyngeal swabs from close contacts to identify asymptomatic respiratory carriers. In view of the erythromycin resistance that had been detected in some strains during outbreak 1, we treated all cases in the second outbreak – symptomatic cases or asymptomatic carriers – with amoxicillin 3 × 1 g (or a combination of amoxicillin plus another agent in case of superinfected wounds) for 14 days. On floor A, two cutaneous diphtheria cases in teenagers were identified on 1 and 23 September, with one of the close contacts, a roommate, being confirmed as an asymptomatic respiratory carrier.

On floor C, the second floor of the centre dedicated to minors, a total of five cases of cutaneous diphtheria were confirmed during medical entry screening. Contact tracing identified one asymptomatic respiratory carrier, who was later confirmed to also have cutaneous diphtheria. Two pharyngeal carriers were identified on floor C, of which one had already been involved in the first outbreak (Case 3 and 17 correspond to the same patient because of reinfection, Supplementary Table S1). Altogether, 140 pharyngeal swabs were taken, and two close contacts of primary cases were confirmed to carry *C*. *diphtheriae*. Residents were offered vaccination within their first week of arrival with an acceptance of over 90%.

Notably, one of the respiratory carriers developed symptoms (tonsillitis), and diphtheria antitoxin (DAT) was administered to prevent further toxin-mediated complications (Case 19, Supplementary Table S1). One minor whose family did not agree to vaccination, and who had been living on another floor than floor C, also developed respiratory symptoms after having left the centre (Case 20, Supplementary Table S1). The individual tested positive for *C. diphtheriae* and required DAT. They were probably infected after contact with one of the minors living on other floors while attending school at the centre.

All patients were male with a median age of 16 years (range 8 to 43 years) and mainly originating from Afghanistan. No information on their medical or vaccination history was available.

### Antimicrobial resistance

**Figure 2:**
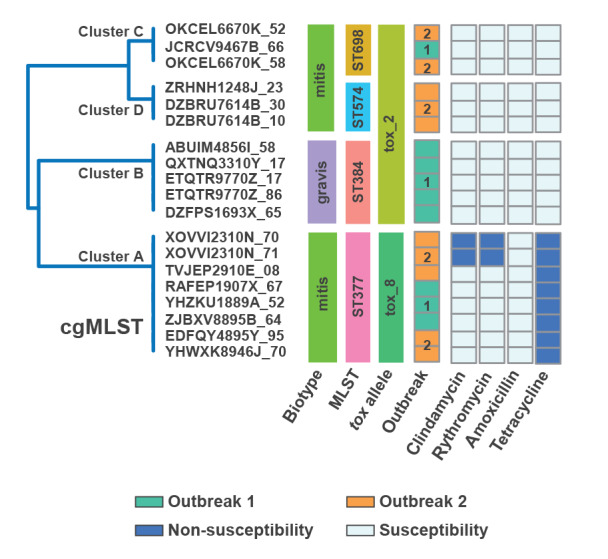
Relationships between cgMLST clusters, *Corynebacterium diphtheriae* biotypes, MLST types, *tox* alleles, outbreak, and antimicrobial resistance profiles of commonly used drugs^a^ to treat *C*. *diphtheriae* infections in two outbreaks at an asylum centre, Switzerland, July–September 2022 (n = 19)

Two *C. diphtheriae* isolates showed unusually broad resistance to many common oral and parenteral antibiotics (ST 377 and ST 698 isolates; Figure 2; predicted antimicrobial resistance genes and phenotypes are shown in the Supplementary Table S3), which made treatment options for bacterial co-infections in wounds challenging. The isolate from Case 10 harboured a class 1 integron carrying a multidrug-resistant gene cassette including *blaOXA-2* (the genomic context of *blaOXA-2* is shown in Supplementary Figure 1), which is a worrisome finding, as class 1 integrons play a major role in the global dissemination of antibiotic resistances via lateral gene transfer into a diversity of bacterial hosts [4].

cgMLST: core genome multi-locus sequence typing; MLST: multi-locus sequence typing.

^a^ Non-susceptibility is indicated in dark blue colour.

### Travel routes

**Figure 3:**
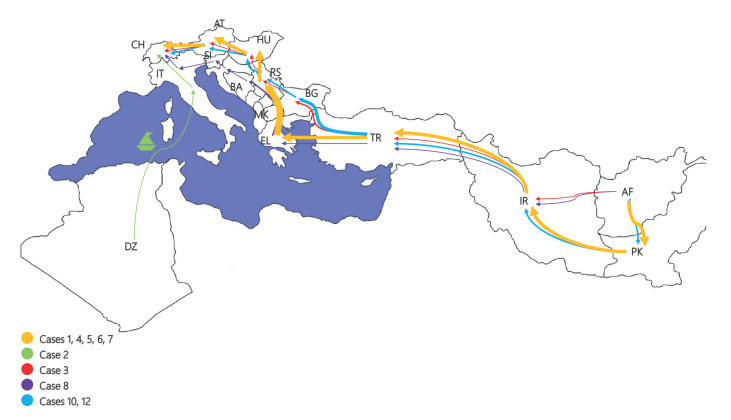
Reported travel routes to Switzerland of *Corynebacterium* *diphtheriae* positive cases in two outbreaks at an asylum centre, Switzerland, July–September 2022 (n = 10)

We performed an epidemiological survey about travel routes which indicated that, while some cases were infected with diphtheria during their stay at the centre, most were probably infected on their journey to Switzerland (Figure 3).

AF: Afghanistan, AT: Austria, BG: Bulgaria, BA: Bosnia and Herzegovina, CH: Switzerland, DZ: Algeria, EL: Greece, HU: Hungary, IR: Iran, IT: Italy, MK: North Macedonia, PK: Pakistan, RS: Serbia, SI: Slovenia, TR: Türkiye.

Data were collected through epidemiological surveys (Supplementary Table S1B).

Data on Cases 9, 11 and 13 to 20 were not available.

Notably, epidemiological surveys revealed that patients often stayed in the same refugee camps in Austria (Vienna area) and Serbia (‘Shamsi’ camp) (Supplementary Table S1B). The latter, along with the whole ‘Balkan route’, was frequently reported as crowded and with poor sanitation facilities, suggesting settings favourable to possible onward transmission.

## Discussion

Diphtheria is an infectious disease caused by Gram-positive bacteria belonging to the genus *Corynebacterium* including the toxigenic species *C. diphtheriae*, which is responsible for the majority of infections in humans. When infecting humans, *C*. *diphtheriae* can lead to respiratory diphtheria or cutaneous diphtheria. Symptomatic infections of toxigenic *C*. *diphtheriae* can lead to toxin-mediated severe complications [5] and the case-fatality rate of diphtheria is 5 to 10% [6].

According to a recent ECDC rapid risk assessment [1] and an update from the WHO/Europe [2], the number of cases reported so far in 2022 is higher than the average number of imported cases seen in recent years. Long-term protection against diphtheria has been shown to be insufficient among migrant populations in Germany in 2015 [7] and in Switzerland in 2019 [8] and cases of diphtheria have been reported among migrant populations in the past [9-14].

Here, we report two outbreaks of diphtheria in Switzerland involving mainly minor asylum seekers who represent a vulnerable population to diphtheria. Most of these minors originate from countries like Afghanistan, where basic health services, including vaccination programmes, are disrupted through long years of conflicts. The 2 years of coronavirus disease (COVID-19) pandemic led to further decreased vaccination coverage [15]. Hence, we can assume that most of them will arrive with absent or incomplete basic immunisations in host countries in Europe, where the overall coverage of the third diphtheria-containing vaccine (DTP3) dose among 1-year-olds varies between 73% and 99% [16]. In Switzerland, coverage reaches 96% [17].

Two cases in our outbreaks had symptomatic respiratory diphtheria and required DAT. After the events described here, two siblings of one of the aforementioned cases, and two additional minor asylum seekers in the centre developed symptoms and required DAT. Therefore, in a short lapse of time of less than 4 weeks, the infection spread to the extent that six minors required DAT therapy. It is the first time since the 1970s that DAT is required in Switzerland.

Genomic analyses evidenced a broad diversity among the isolates from the cases in the centre at the levels of *C. diphtheriae* biotypes, MLST, cgMLST and single nucleotide polymorphisms profiles (Figure 1 and Figure 2), inconsistent with a scenario of a single spreading event of an epidemic clone. Instead, a more complex situation was revealed with genomic clusters shared across three residence floors, and also up to three among four MLST types (ST 377, ST 384, ST 698, ST 574) circulating on the same floor. Those MLST types have already been documented in various countries, as reported by the ECDC [1]. Our genomic analysis involved at least five strains (one of which *C. phoceense*) and showed no monoclonality. This information combined with the analysis of the travel routes suggests that a minority of cases were infected during their stay at our centre, and most seem to have been infected on their journey to Switzerland.

Although data presented here are limited by the relatively small number of cases, especially regarding the analysis of travel routes, the cases involved in the continuing outbreak are consistent with the data collected here. By 28 October, the second outbreak involved 25 patients at the centre, 23 of them being teenagers from Afghanistan.

Our data reveal a higher carriage rate of diphtheria in our asylum seeker setting than anticipated. We propose countries, in particular those along the common migration routes, to consider intensifying or implementing public health measures. Such measures should consist of a medical entry screening, especially for minor asylum seekers, including systematic collection of pharyngeal swabs and swabs of (even the smallest) wounds, prophylactic and therapeutic treatment as appropriate, a proactive offer of vaccination, and cohorting of residents at entry, until the results of swabs are received. In order to optimise protection against vaccine-preventable diseases, all people working in settings with asylum seekers, in particular with minors, are highly recommended to keep their vaccinations up to date.

### Conclusions

The diphtheria outbreak at the centre in Bern is still ongoing at the time of publication, reflecting the difficulty to stop the spread of *C. diphtheriae* inside a facility where many people with low vaccination coverage live under crowded conditions. By 28 October, six patients required DAT therapy. Vaccination as well as screening for and, if necessary, treatment of diphtheria, should be done with focus on unaccompanied minors and immediately upon entry into Europe. Epidemiological data revealed similar migration routes that should guide these public health measures.

## Supplementary Data

397000 Supplementary Material
