# Supplementary material for: Ongoing toxin-positive diphtheria outbreaks in a federal asylum centre in Switzerland, analysis July to September 2022
Source: Euro Surveill. 2022 Nov 3;27(44):2200811. doi: 10.2807/1560-7917.ES.2022.27.44.2200811 (PMC9635023; doi:10.2807/1560-7917.ES.2022.27.44.2200811)
Supplement: Supplementary Material [file 22-00811_GRUTZMACHER_Supplementary_material.pdf]

Supplementary material of “Ongoing toxin-positive diphtheria outbreaks in a federal asylum centre in Switzerland, analysis July to September 2022”

This supplementary material is hosted by *Eurosurveillance* as supporting information alongside the article ‘Ongoing toxin-positive diphtheria outbreaks in a federal asylum centre in Switzerland, analysis July to September 2022’, on behalf of the authors, who remain responsible for the accuracy and appropriateness of the content. The same standards for ethics, copyright, attributions and permissions as for the article apply. Supplements are not edited by *Eurosurveillance* and the journal is not responsible for the maintenance of any links or email addresses provided therein.

Case descriptions

Altogether 19 patients (20 cases, of which one was a reinfection) had positive swabs for *C. diphtheriae* in either respiratory or cutaneous specimens, of which 17 had positive swabs for toxin-positive *C. diphtheriae* (Supplementary Table S1A). All patients were male with a median age of 16 years (range 8 to 43 years). No information on their medical or vaccination history was available. During the first outbreak, most residents received vaccination after the beginning of the outbreak, while in the second outbreak residents had already been vaccinated soon after their arrival. Patients with cutaneous diphtheria were treated with amoxicillin for 14 days (1 g tid) or co-amoxicillin (1 g tid) resp. another antibiotic combination for 14 days in case of wound co-infection with other bacterial organisms (mainly *Streptococcus* spp. and *Staphylococcus aureus*, including methicillin-resistant *S. aureus*). Asymptomatic carriers received azithromycin 500 mg OD for three days in the first outbreak. In the second outbreak all positive cases (irrespective of symptoms or not) were treated with amoxicillin 3x1g for 14 days (or a combination of amoxicillin plus another agent in case of superinfected wounds) since some strains from outbreak 1 showed resistance to macrolides.

Genomic library preparation and Whole-genome sequencing

Genomic DNA was purified from bacterial cultures using PureLink Genomic DNA kit (Thermofisher, Switzerland). NGS libraries were prepared using Illumina Nextera DNA Flex Library Prep kit (Illumina, Switzerland) and sequenced using an Illumina MiSeq benchtop sequencer generating 2 ×150 bp paired-end reads (v2), according to the manufacturer’s protocols, at the Next Generation Sequencing Platform of the Institute for Infectious Diseases, Bern.

Bioinformatic analyses

After read quality checking using FastQC (v0.11.8; https://www.bioinformatics.babraham.ac.uk/), paired-end reads were quality-filtered, trimmed and *de novo* assembled using *shovill* (v1.0.4; default: SPAdes; https:// github.com/ tseemann/shovill). Reads and genome assemblies were deposited at NCBI under BioProject ID PRJNA889706.

Genome annotation was performed in the framework of the Reference Sequence (*RefSeq*) project using *Prokaryotic Genome Annotation Pipeline* (*PGAP*) [1] at the National Center for Biotechnology Information (NCBI). The quality of genome sequences recovered from the isolates was assessed for genome completeness and contamination by using collocated sets of genes that are ubiquitous and single-copy within a phylogenetic lineage using *CheckM (v*1.0.18, [2]), and by using *MetaPhlAn2* (Metagenomic Phylogenetic Analysis) (v2.7.7 [3]), which profiles the composition of microbial communities from metagenomic shotgun sequencing data based on unique clade-specific marker genes identified from ~17,000 reference genomes.

Recruitment of the most closely related genomes was done by submitting the genome assemblies to *KmFinder (v3.2; https://cge.food.dtu.dk/services/KmerFinder/* [4]) and computing the "mash dist" from sketches of all *Corynebacterium* genomes in NCBI RefSeq

(https://www.ncbi.nlm.nih.gov/refseq/; download date: 2022-08-03). The overall closest assigned *C. diphtheriae* genome sequences with known biotype, as well as the *C. diphtheriae* and *C. phoceense* reference genomes were selected for Average Nucleotide Identities (ANI) comparisons. We used ANI with MUMmer (NUCmer; ANIm) to align the input sequences. ANI is proposed to be the appropriate *in silico* substitute for DNA-DNA hybridisation (DDH), and may be useful for delineating species boundaries. A typical percentage threshold for species boundary in the literature is 95% ANI (e.g. [5]). Biotype mitis vs. gravis was determined and by identifying the gravis-specific SNP [6] at position 313818 (reference NCTC13129) with *snippy* (v4.6.0, https://github.com/tseemann/snippy) and *BLASTn* (v2.10.1) similarity search against the *spuA* gene, which codes for a putative alpha-1,6-glycosidase which may be specific for biovar gravis isolates. Evaluation of drug-resistance profiles were predicted by ResFinder (v4.1 server, default parameters, https://cge.food.dtu.dk/services/ResFinder/ [4]).

Core genome multilocus sequence typing (cgMLST) was used to delineate pathogen transmission clusters using a gene-by-gene allele calling approach on Ridom SeqSphere v8.4.0 software (Ridom, Munster, Germany). All of the genome assemblies for each of the samples were BLASTn aligned to the cgMLST scheme, which was retrieved from the Institut Pasteur scheme (https://bigsdb.pasteur.fr/cgi-bin/bigsdb/bigsdb.pl?db=pubmlst_diphtheria_seqdef&page=downloadAlleles) encompassing 1319 core genes, including the seven genes used for MLST typing (839 profiles), using default SeqSphere quality and matching parameters. Unweighted Pair Group Method with Arithmetic Mean (UPGMA) dendrogram was drawn based on the number of allele differences across genomes. Similarly, the FASTA sequences for the *tox* gene (38 allelic profiles) were downloaded and imported into SeqSphere for identifying and typing the diphtheria toxin gene. cgMLST clusters A, B, C, D were defined *ad hoc*, and were separated by the following number of allele differences [B-(935)-A-(1049)-D-(267)-C] in a minimum spanning tree (MST) representation of the allelic distance matrix (data not shown).

Thus, to obtain a finer resolution of transmission events within the four cgMLST clusters, Single Nucleotide Polymorphism (SNP) analysis between genome sequences was performed by considering the sequence of the first isolate in each cgMLST cluster as reference for the rest of the cgMLST cluster, and using *snippy* (default parameters) and genome annotation with *prokka (v. 1.14.0,* [7] for the genomic comparison.

Based on the estimate of rate of molecular evolution of 1.67×10^{-6} substitutions per site per year for *C. diphtheriae* [8], and considering an average genome size of 2,500,000 bp and one year consists of 52.14 weeks on average, we estimated that one observed mutation between any two genome sequences (Suppl. Table S2) may have appeared not before six weeks after transmission, and that 2 SNPs may have appeared after 5 months (19 weeks), 5 SNPs after 1.1 years (57 weeks), and 10 SNPs after 2.3 years (119 weeks). Additional graphics and analyses were produced in R with the libraries *dplyr, reshape2*, *ggplot2*, *ggnetwork*, *ape*, *pheatmap* and *igraph.*

Genomic context of *blaOXA-2* in XOVVI2310N_71

In XOVVI2310N_71, the *blaOXA-2* gene was located on a class 1 integron containing in addition aminoglycoside adenyltransferase (*aadA1*), truncated quaternary ammonium compound-resistance protein (*qacE*), with high similarity to MDR Pseudomonas aeruginosa strain 85-7090A integron (Supplementary Figure 1) and to integrons carrying MDR gene cassettes found in other Gram-positive and Gram-negative MDR bacteria in general (data not shown). Class 1 integrons play a major role in the global dissemination of antibiotic resistances via lateral gene transfer into a diversity of bacterial hosts.

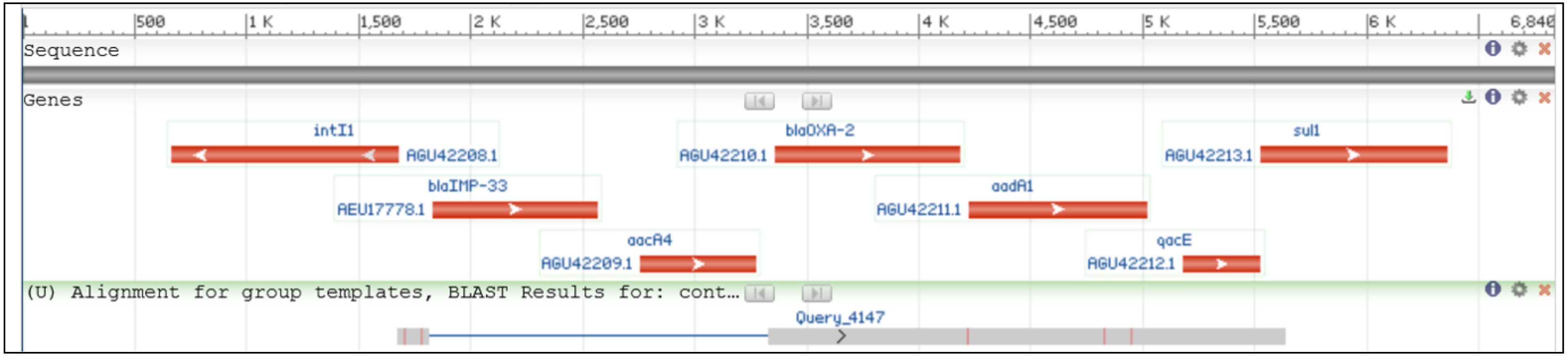

Supplementary Figure 1: Genomic context of *blaOXA-2* in isolate XOVVI2310N_71. Pairwise BLAST (blastn) was performed online (query_4147; https://blast.ncbi.nlm.nih.gov) against *Pseudomonas aeruginosa* strain 85-7090A Class I integron sequence that harbors integrase (*intI1*), metallo-beta-lactamase IMP-33 (*blaIMP-33*), aminoglycoside acetyltransferase (*aacA4*), ESBL oxacillinase (*blaOXA-2*), aminoglycoside adenyl transferase (*aadA1*), truncated quaternary ammonium compound-resistance protein (*qacE*), and sulphonamide resistance determinant (*sul1*) genes (GenBank accession JN848782.2).

Supplementary Tables

Supplementary Table S1A: Table of cases with the pre-treatment course. FAC: Federal Asylum Center, N/A: Not available, ≠ : No data, WGS: Whole genome sequencing.

ID		Demographical data					Cutaneous swab results				Elek test / WGS on cutaneous culture		Pharyngeal swab results			Elek test / WGS* on pharyngeal culture	
Out-break	Case	Age	Floor	Country of origin	Entry in FAC	Isolation date	Collection date	Wound	Date of result	Result	Date of result	Result	Collection date	Date of result	Result	Date of result	Result
1	1	<18	A	Afghanistan	Mid of July 2022	21.07.22	18.07.22	Left tibia	21.07.22	positive	25.07.22	positive	21.07.22	26.07.22	positive	08.08.22	positive
1	2	20 - 30	B	Algeria	Mid of July 2022	26.07.22	22.07.22	Lower leg	26.07.22	positive	28.07.22	negative	26.07.22	28.07.22	positive	01.08.22	positive
1	3	<18	A	Afghanistan	End of July 2022	26.07.22	25.07.22	Both legs	26.07.22	positive	29.07.22	positive	27.07.22	29.07.22	negative	≠	≠
1	4	<18	A	Afghanistan	Mid of July 2022	30.07.22	≠	≠	≠	≠	≠	≠	27.07.22	29.07.22	positive	03.08.22	positive
1	5	<18	A	Afghanistan	Mid of July 2022	30.07.22	≠	≠	≠	≠	≠	≠	27.07.22	29.07.22	positive	03.08.22	positive
1	6	<18	A	Afghanistan	Mid of July 2022	30.07.22	≠	≠	≠	≠	≠	≠	27.07.22	29.07.22	positive	03.08.22	positive
1	7	<18	A	Afghanistan	Mid of July 2022	30.07.22	≠	≠	≠	≠	≠	≠	27.07.22	29.07.22	positive	03.08.22	positive
1	8	20 - 30	B	Afghanistan	End of July 2022	03.08.22	29.07.22	Right finger	03.08.22	positive	05.08.22	positive	02.08.22	04.08.22	negative	≠	≠
2	9	<18	A	Afghanistan	End of August 2022	01.09.22	30.08.22	Right foot	01.09.22	positive	05.09.22	positive	01.09.22	05.09.22	positive	09.09.22	positive
2	10	<18	C	Afghanistan	End of August 2022	05.09.22	02.09.22	Both hands	05.09.22	positive	08.09.22	positive	05.09.22	07.09.22	positive	08.09.22	positive

2	11	<18	A	Afghanistan	Beginning of September 2022	07.09.22	08.09.22	Left calves	12.09.22	negative	≠	≠	05.09.22	07.09.22	positive	08.09.22	negative*
2	12	<18	C	Afghanistan	Mid of August 2022	09.09.22	07.09.22	Foot	09.09.22	positive	12.09.22	positive*	09.09.22	12.09.22	positive	15.09.22	positive*
2	13	<18	C	Afghanistan	Mid of September 2022	13.09.22	12.09.22	Right knee, both feet	13.09.22	positive	15.09.22	positive*	13.09.22	15.09.22	negative	≠	≠
2	14	<18	C	Afghanistan	Mid of September 2022	15.09.22	14.09.22	Right foot	17.09.22	positive	19.09.22	positive*	14.09.22	15.09.22	positive	16.09.22	positive*
2	15	40 - 50	C	Pakistan	Mid of September 2022	21.09.22	20.09.22	Right foot	21.09.22	positive	23.09.22	negative*	21.09.22	23.09.22	negative	≠	≠
2	16	<18	A	Afghanistan	Mid Of September 2022	23.09.22	22.09.22	Left foot	23.09.22	positive	26.09.22	positive*	22.09.22	24.09.22	negative	≠	≠
2	17	<18	C	Afghanistan	End of July 2022	27.09.22	≠	≠	≠	≠	≠	≠	26.09.22	28.09.22	positive	28.09.22	positive*
2	18	<18	C	Afghanistan	End of September 2022	28.09.22	27.09.22	Left foot	28.09.22	positive	03.10.22	positive*	27.09.22	28.09.22	positive	03.10.22	positive*
2	19	<18	C	Afghanistan	End of June 2022	28.09.22	≠	≠	≠	≠	≠	≠	26.09.22	28.09.22	positive	26.09.22	positive*
2	20	<10	N/A	Russia	Mid of August 2022	23.09.22	≠	≠	≠	≠	≠	≠	21.09.22	23.09.22	positive	14.10.22	positive*

* Toxigenicity tested by WGS.

Supplementary Table S1B: Table of cases with indicated antibiotic treatment, control swabs and geographical data. Antibiotic treatment consisted of either Amoxicillin (500 mg), co-Amoxicillin (1000 mg) or Azithromycin (500 mg). FAC: Federal Asylum Center, N/A: Not available. Epidemiological data were collected after patients gave their informed consent.

ID		Antibiotic therapy or prophylaxis			First control pharyngeal swab			Second control pharyngeal swab			Isolation	Geographical data		
Out-break	Case	Start	Antibiotics	End	Date of control swab	Date of result	Result	Date of control swab	Date of result	Result	Date end of isolation	Date of application for asylum	Location of application for asylum	Camp stays
1	1	21.07.22	Amoxicillin	04.08.22	05.08.22	08.08.22	negative	08.08.22	10.08.22	negative	10.08.22	Mid of July 2022	Boudry	Serbia and Vienna
1	2	26.07.22	co-Amoxicillin	09.08.22	10.08.22	12.08.22	negative	11.08.22	13.08.22	negative	15.08.22	Mid of July 2022	Zurich	None
1	3	26.07.22	Amoxicillin	09.08.22	11.08.22	15.08.22	negative	10.08.22	12.08.22	negative	15.08.22	Mid of July 2022	Zurich	Serbia (“Shamsi”) and Austria
1	4	27.07.22	Azithromycin	29.07.22	01.08.22	04.08.22	negative	02.08.22	04.08.22	negative	04.08.22	Mid of July 2022	Zurich	Serbia (“Shamsi”) and Vienna
1	5	27.07.22	Azithromycin	29.07.22	01.08.22	04.08.22	negative	02.08.22	04.08.22	negative	04.08.22	Mid of July 2022	Zurich	Vienna
1	6	27.07.22	Azithromycin	29.07.22	01.08.22	04.08.22	negative	02.08.22	04.08.22	negative	04.08.22	Mid of July 2022	Zurich	Serbia and Austria
1	7	27.07.22	Azithromycin	29.07.22	01.08.22	04.08.22	negative	02.08.22	04.08.22	negative	04.08.22	Mid of July 2022	Zurich	Serbia (“Berinas”) and Austria
1	8	03.08.22	co-Amoxicillin	16.08.22	17.08.22	19.08.22	negative	18.08.22	20.08.22	negative	22.08.22	Mid of July 2022	Zurich	Serbia (“Prishio”)
2	9	01.09.22	co-Amoxicillin	15.09.22	16.09.22	19.09.22	negative	19.09.22	21.09.22	negative	21.09.22	End of August 2022	Zürich	N/A
2	10	05.09.22	co-Amoxicillin	19.09.22	20.09.22	22.09.22	negative	21.09.22	23.09.22	negative	23.09.22	End of August 2022	Zurich	Serbia (“Shamsi”) and Vienna
2	11	05.09.2022<br>07.09.2022	Azithromycin<br>Amoxicycline	07.09.2022<br>21.09.2022	12.09.22	14.09.22	negative	13.09.22	15.09.22	negative	15.09.22	Beginning of September 2022	Bern	N/A

2	12	09.09.22	co-Amoxicillin	23.09.22	26.09.22	28.09.22	negative	27.09.22	29.09.22	negative	29.09.22	Mid of August 2022	Zurich	Serbia (“Shamsi”) and Vienna
2	13	13.09.22	co-Amoxicillin	27.09.22	28.09.22	30.09.22	negative	29.09.22	01.10.22	negative	03.10.22	Mid of September 2022	Zürich	N/A
2	14	14.09.22<br>15.09.22	Azithromycin<br>co-Amoxicycline	15.09.22<br>29.09.22	30.09.22	03.10.22	negative	03.10.22	05.10.22	negative	05.10.22	Mid of September 2022	Zürich	N/A
2	15	21.09.22	Amoxicilline	06.10.22	07.10.22	10.10.22	negative	10.10.22	12.10.22	negative	12.10.22	Mid of September 2022	Zürich	N/A
2	16	23.09.22	co-Amoxicillin	08.10.22	11.10.22	13.11.22	negative	12.10.22	14.10.22	negative	14.10.22	Mid of September 2022	Zürich	N/A
2	17	28.09.22	Amoxicillin	05.10.22	06.10.22	10.10.22	negative	07.10.22	10.10.22	negative	10.10.22	Mid of September 2022	Zürich	N/A
2	18	29.09.22	co-Amoxicillin	13.10.22	14.10.22	17.10.22	negative	17.10.22	19.10.22	negative	19.10.22	Mid of September 2022	Zurich	N/A
2	19	26.09.22	Amoxicillin	10.10.22	11.10.22	13.10.22	negative	12.10.22	14.10.22	negative	14.10.22	Mid of June 2022	Zurich	N/A
2	20	23.09.22	Amoxicillin	ongoing	06.10.22	07.10.22	positive	20.10.22	21.10.22	positive	N/A	Mid of August 2022	Bern	N/A

Supplementary Table S2: Number of SNP differences as compared to the reference case (in bold font). Only the lower values of each matrix are shown. Ref: Reference.

ClusterA

	cases	7	6	10	10	13	14	16
ZJBXV8895B_64	7							
RAFEP1907X_67	6	4						
XOVVI2310N_71	10	164	162					
XOVVI2310N_70	10	163	161	1				
YHWXK8946J_70	13	4	2	162	161			
EDFQY4895Y_95	14	5	3	163	162	3		
TVJEP2910E_08	16	8	6	164	163	6	7	
**YHZKU1889A_52**	**3 (Ref)**	10	8	164	163	8	9	10

ClusterB

	cases	**1 (Ref)**	1	2	5
**ETQTR9770Z_17**	**1 (Ref)**				
ETQTR9770Z_86	1	1			
ABUIM4856I_58	2	5	6		
DZFPS1693X_65	5	0	1	5	
QXTNQ3310Y_17	8	0	1	5	0

ClusterC

	cases	**4 (Ref)**	9
**JCRCV9467B_66**	**4 (Ref)**		
OKCEL6670K_58	9	0	
OKCEL6670K_52	9	1	1

ClusterD

		**12 (Ref)**	12	17
**DZBRU7614B_30**	**12 (Ref)**			
DZBRU7614B_10	12	4		
ZRHNH1248J_23	17	5	9	

Supplementary Table S3: Predicted antimicrobial resistance genes and phenotypes. R: Resistant, S: Susceptible.

	amoxicillin	amoxicillin+clavulanic acid	piperacillin+tazobactam	ceftazidime	chloramphenicol	erythromycin	clindamycin	doxycycline	tetracycline	minocycline	fluoroquinolone	gentamicin	tobramycin	streptomycin	sulfamethoxazole	trimethoprim	MLST	Cluster
ETQTR9770Z_17	S	S	S	S	S	S	S	S	S	S	S	S	S	S	S	S	ST 384	B
ETQTR9770Z_86	S	S	S	S	S	S	S	S	S	S	S	S	S	S	S	S	ST 384	B
ABUIM4856I_02	S	S	S	S	R (2)	R (1)	R (1)	S	S	S	S	S	S	R (3)	S	S	*C. phoceense*	
YHZKU1889A_52	S	S	S	S	R (2)	S	S	R (2)	R (2)	S	S	S	S	R (3)	R (1)	R (3)	ST 377	A
ABUIM4856I_58	S	S	S	S	S	S	S	S	S	S	S	S	S	S	S	S	ST 384	B
ZJBXV8895B_64	S	S	S	S	R (2)	S	S	R (2)	R (2)	S	S	S	S	R (3)	R (1)	R (3)	ST 377	A
DZFPS1693X_65	S	S	S	S	S	S	S	S	S	S	S	S	S	S	S	S	ST 384	B
JCRCV9467B_66	S	S	S	S	R (2)	S	S	S	S	S	S	S	S	R (3)	R (3)	S	ST 698	C
RAFEP1907X_67	S	S	S	S	R (2)	S	S	R (2)	R (2)	S	S	S	S	R (3)	R (1)	R (3)	ST 377	A
QXTNQ3310Y_17	S	S	S	S	S	S	S	S	S	S	S	S	S	S	S	S	ST 384	B
OKCEL6670K_58	S	S	S	S	R (2)	S	S	S	S	S	S	S	S	R (3)	R (3)	S	ST 698	C
OKCEL6670K_52	S	S	S	S	R (2)	S	S	S	S	S	S	S	S	R (3)	R (3)	S	ST 698	C
XOVVI2310N_71	R (3)	R (3)	S	R (3)	S	R (1)	R (1)	R (2)	R (2)	R (2)	S	R (3)	R (3)	R (3)	R (2)	R (3)	ST 377	A
GVUBY4395B_94	S	S	S	S	R (2)	S	S	S	S	S	S	S	S	R (3)	R (3)	S		
XOVVI2310N_70	R (3)	R (3)	S	R (3)	S	R (1)	R (1)	R (2)	R (2)	R (2)	S	R (3)	R (3)	R (3)	R (2)	R (3)	ST 377	A
DZBRU7614B_30	S	S	S	S	R (2)	S	S	S	S	S	S	S	S	R (3)	R (3)	S	ST 574	D
DZBRU7614B_10	S	S	S	S	R (2)	S	S	S	S	S	S	S	S	R (3)	R (3)	S	ST 574	D
YHWXK8946J_70	S	S	S	S	R (2)	S	S	R (2)	R (2)	S	S	S	S	R (3)	R (1)	R (3)	ST 377	A
EDFQY4895Y_95	S	S	S	S	R (2)	S	S	R (2)	R (2)	S	S	S	S	R (3)	R (1)	R (3)	ST 377	A
NDZRZ6050V_23	S	S	S	S	R (2)	R (1)	R (1)	R (2)	R (2)	R (2)	S	S	S	S	S	S	ST103	
TVJEP2910E_08	S	S	S	S	R (2)	S	S	R (2)	R (2)	S	S	S	S	R (3)	R (1)	R (3)	ST 377	A
ZRHNH1248J_23	S	S	S	S	R (2)	S	S	S	S	S	S	S	S	R (3)	R (3)	S	ST 574	D

Numbers in parentheses indicate the degree of sequence match for a given resistance gene (ResFinder) with:
(1) = Match < 100% identity AND match length < reference length
(2) = Match = 100% identity AND match length < reference length
(3) = Match = 100% identity AND match length = reference length

All predicted susceptibility data indicated in green were confirmed by phenotype assays. Those marked in red do not match phenotype results.

Supplementary Table S4: GenBank accession numbers for the sequenced genome sequences in this study (BioProject ID PRJNA889706).

Sample N ame	Accession	Organism	URLs
ETQTR9770Z_17	SAMN31250445	Corynebacterium diphtheriae	https://www.ncbi.nlm.nih.gov/sra/31250445
ETQTR9770Z_86	SAMN31250446	Corynebacterium diphtheriae	https://www.ncbi.nlm.nih.gov/sra/31250446
ABUIM4856I_02	SAMN31250447	Corynebacterium phoceense	https://www.ncbi.nlm.nih.gov/sra/31250447
ABUIM4856I_58	SAMN31250448	Corynebacterium diphtheriae	https://www.ncbi.nlm.nih.gov/sra/31250448
YHZKU1889A_52	SAMN31250449	Corynebacterium diphtheriae	https://www.ncbi.nlm.nih.gov/sra/31250449
JCRCV9467B_66	SAMN31250450	Corynebacterium diphtheriae	https://www.ncbi.nlm.nih.gov/sra/31250450
DZFPS1693X_65	SAMN31250451	Corynebacterium diphtheriae	https://www.ncbi.nlm.nih.gov/sra/31250451
RAFEP1907X_67	SAMN31250452	Corynebacterium diphtheriae	https://www.ncbi.nlm.nih.gov/sra/31250452
ZJBXV8895B_64	SAMN31250453	Corynebacterium diphtheriae	https://www.ncbi.nlm.nih.gov/sra/31250453
QXTNQ3310Y_17	SAMN31250454	Corynebacterium diphtheriae	https://www.ncbi.nlm.nih.gov/sra/31250454
OKCEL6670K_58	SAMN31250455	Corynebacterium diphtheriae	https://www.ncbi.nlm.nih.gov/sra/31250455

OKCEL6670K_52	SAMN31250456	Corynebacterium diphtheriae	https://www.ncbi.nlm.nih.gov/sra/31250456
XOVVI2310N_71	SAMN31250457	Corynebacterium diphtheriae	https://www.ncbi.nlm.nih.gov/sra/31250457
XOVVI2310N_70	SAMN31250458	Corynebacterium diphtheriae	https://www.ncbi.nlm.nih.gov/sra/31250458
GVUBY4395B_94	SAMN31250459	Corynebacterium diphtheriae	https://www.ncbi.nlm.nih.gov/sra/31250459
DZBRU7614B_30	SAMN31250460	Corynebacterium diphtheriae	https://www.ncbi.nlm.nih.gov/sra/31250460
DZBRU7614B_10	SAMN31250461	Corynebacterium diphtheriae	https://www.ncbi.nlm.nih.gov/sra/31250461
YHWXK8946J_70	SAMN31250462	Corynebacterium diphtheriae	https://www.ncbi.nlm.nih.gov/sra/31250462
EDFQY4895Y_95	SAMN31250463	Corynebacterium diphtheriae	https://www.ncbi.nlm.nih.gov/sra/31250463
NDZRZ6050V_23	SAMN31250464	Corynebacterium diphtheriae	https://www.ncbi.nlm.nih.gov/sra/31250464
TVJEP2910E_08	SAMN31250465	Corynebacterium diphtheriae	https://www.ncbi.nlm.nih.gov/sra/31250465
ZRHNH1248J_23	SAMN31250466	Corynebacterium diphtheriae	https://www.ncbi.nlm.nih.gov/sra/31250466

References

1. Haft, D.H., M. DiCuccio, A. Badretdin, V. Brover, V. Chetvernin, K. O'Neill, W. Li, F. Chitsaz, M.K. Derbyshire, N.R. Gonzales, M. Gwadz, F. Lu, G.H. Marchler, J.S. Song, N. Thanki, R.A. Yamashita, C. Zheng, F. Thibaud-Nissen, L.Y. Geer, A. Marchler-Bauer, and K.D. Pruitt, RefSeq: an update on prokaryotic genome annotation and curation. Nucleic Acids Res, 46(D1): p. D851-D860, 2018

2. Parks, D.H., M. Imelfort, C.T. Skennerton, P. Hugenholtz, and G.W. Tyson, CheckM: assessing the quality of microbial genomes recovered from isolates, single cells, and metagenomes. Genome Res, 25(7): p. 1043-1055, 2015

3. Segata, N., L. Waldron, A. Ballarini, V. Narasimhan, O. Jousson, and C. Huttenhower, Metagenomic microbial community profiling using unique clade-specific marker genes. Nat Methods, 9(8): p. 811-814, 2012

4. Hasman, H., D. Saputra, T. Sicheritz-Ponten, O. Lund, C.A. Svendsen, N. Frimodt-Moller, and F.M. Aarestrup, Rapid whole-genome sequencing for detection and characterization of microorganisms directly from clinical samples. J Clin Microbiol, 52(1): p. 139-146, 2014

5. Richter, M. and R. Rossello-Mora, Shifting the genomic gold standard for the prokaryotic species definition. Proc Natl Acad Sci U S A, 106(45): p. 19126-19131, 2009

6. Hennart, M., L.G. Panunzi, C. Rodrigues, Q. Gaday, S.L. Baines, M. Barros-Pinkelnig, A. Carmi-Leroy, M. Dazas, A.M. Wehenkel, X. Didelot, J. Toubiana, E. Badell, and S. Brisse, Population genomics and antimicrobial resistance in Corynebacterium diphtheriae. Genome Medicine, 12(1): p. 107, 2020

7. Seemann, T., Prokka: rapid prokaryotic genome annotation. Bioinformatics, 30(14): p. 2068-2069, 2014

8. Badell, E., A. Alharazi, A. Criscuolo, K.A.A. Almoayed, N. Lefrancq, V. Bouchez, J. Guglielmini, M. Hennart, A. Carmi-Leroy, N. Zidane, M. Pascal-Perrigault, M. Lebreton, H. Martini, H. Salje, J. Toubiana, F. Dureab, G. Dhabaan, S. Brisse, A.A. Rawah, M.A. Aldawla, E.M. Al-Awdi, N.M. Al-Moalmy, H.Z. Al-Shami, and A.A. Al-Somainy, Ongoing diphtheria outbreak in Yemen: a cross-sectional and genomic epidemiology study. The Lancet Microbe, 2(8): p. e386-e396, 2021
